# Beyond Immunosuppression: Defining Perioperative Risk in Transplant Patients Undergoing Colorectal and Small Bowel Surgery

**DOI:** 10.1002/wjs.70285

**Published:** 2026-03-04

**Authors:** Phil Meister, Samira Vestweber, Jan Neuhaus, Marc A. Reschke, Ulf Neumann, Andreas D. Rink

**Affiliations:** ^1^ Department of General‐, Visceral‐, Vascular‐ and Transplantation Surgery University Hospital Essen Essen Germany

**Keywords:** colorectal cancer, immunosuppressant, surgery, transplantation

## Abstract

**Background:**

Colorectal and small bowel surgery in transplant (TX) recipients presents unique perioperative challenges due to immunosuppression and comorbidities, with poorly defined risks.

**Methods:**

A retrospective analysis was conducted on 237 TX recipients who underwent colorectal or small bowel surgery at a specialized center between 2008 and 2024. Patient characteristics (transplant type, time since TX, immunosuppression, Charlson Comorbidity Index) and surgical details were analyzed in relation to postoperative outcomes (ICU stay, in‐hospital mortality, length of stay, major morbidity [Dindo‐Clavien ≥ 3]).

**Results:**

Most patients had prior kidney (45.6%) or liver TX (28.3%). The incidence of all adverse endpoints was significantly higher in emergencies as compared to elective surgery (mortality 25.6% vs. 6.2%, morbidity 52.3% vs. 35.4%, LOS 24.9d vs. 15.7d, ICU stay 10.7d vs. 2.9d; all *p* ≤ 0.001). 14.8% had surgical site infections, 9.7% cardiopulmonary complications. Primary anastomosis was not correlated with worse outcome. Multivariate regression showed emergency surgery (OR 8.48 (2.68–26.8) *p* = 0.001), colorectal surgery (OR 3.26 (1.54–6.90) *p* = 0.002) and heart TX (OR 4.35 (1.10–17.23) *p* = 0.036) as independent risk factors, patients receiving prednisolone had reduced risk (OR 0.28 (0.13–0.60) *p* = 0.001). Heart TX and emergency surgery correlated with longer ICU stay, hematopoetic stem cell transplantation with longer LOS.

**Conclusions:**

TX recipients undergoing colorectal or small bowel surgery face considerable perioperative risks, especially in emergency situations and after heart transplantation. The feasibility of primary anastomosis in selected patients suggests that surgical strategies should be tailored, emphasizing the need for specialized, multidisciplinary care to optimize outcomes in this vulnerable population.

## Highlights

*What does this paper add to the literature?* This paper reports of the largest cohort of transplant recipients undergoing intestinal surgery. It concretizes perioperative risk and identifies heart transplantation recipients at particular risk. Morbidity consists not only of surgical site infections but also of a relevant number of cardiopulmonary complications.

## Graphical abstract

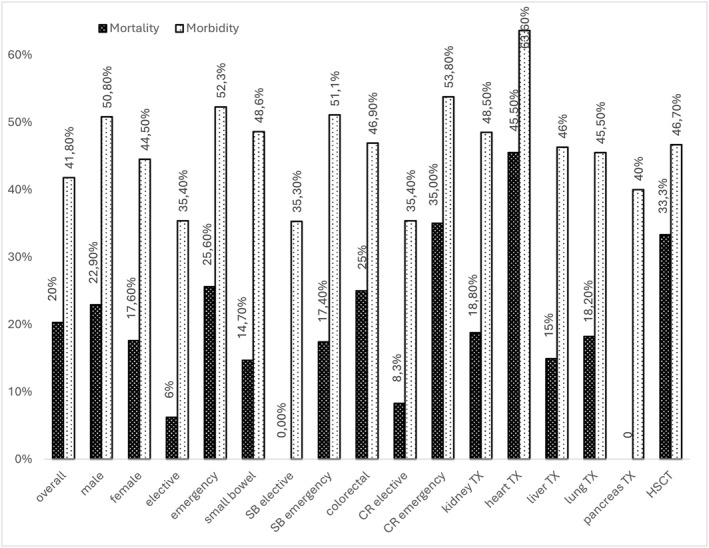

**Abbreviations**
CCI: Charlson Comorbidity Index
CRC: Colorectal Cancer
HSCT: hematopoietic stem cell transplantation
ICU: Intensive Care Unit
OR: Odds Ratio
SSI: Surgical Site Infection
TX: Transplantation

## 1 Introduction

Transplant (TX) recipients present a unique surgical risk profile. Immunosuppression, while necessary, can predispose these patients to postoperative infections and impaired wound healing. However, the actual operative risk remains unclear and comprehensive analysis is needed.

Large registry studies have reported poorer postoperative outcomes in kidney transplant recipients [1, 2]. Limited data exist for specific disease entities in this population. For example, acute appendicitis is associated with a 25% morbidity rate in a series of 17 TX patients [3]. The US transplant registry documented increased mortality and morbidity in heart transplant recipients with acute cholecystitis [4]. A small series of 7 heart or lung transplant patients undergoing complex biliary surgery reported a 29% mortality rate. [5]. Similarly, a case‐control study of cholecystitis in 32 hematopoietic stem cell transplant (HSCT) recipients revealed a 15% perioperative mortality rate [6]. Pancreatic surgery in liver transplant recipients, as reported in two series of 9 and 18 patients, demonstrated major morbidity rates of 22% and 77.8%, respectively [7, 8].

Acute diverticulitis has been more extensively studied in TX patients. Initial reports highlighted a significantly elevated risk of colectomy [9, 10, 11]. Consequently more recent evidence suggests that conservative management is safe and feasible in complicated acute diverticulitis in immunocompromised patients [12, 13, 14]. In accordance to these findings, treatment recommendations for diverticulitis in TX patients have changed to a less aggressive surgical approach. On the other hand, a joint guideline for acute abdomen in immunocompromised patients has been published in 2021, where despite acknowledging the increased perioperative risk, surgery remains the standard treatment for appendicitis and cholecystitis due to a lack of robust data on alternative therapies [15].

Beyond acute pathologies, cancer development, particularly colorectal cancer (CRC), poses a significant challenge in TX patient management. CRC exhibits a more aggressive phenotype and is associated with poorer outcomes in this population [16, 17]. A recent case‐matched study [18] demonstrated an increased risk of CRC surgery in TX patients. However, chemotherapy and other systemic treatments also present unique challenges in this cohort [19].

This fragmentary data landscape might lead to uncertainty and apprehension, already resulting in TX patients with CRC being less likely to receive adequate treatment [20].

Therefore, this study aims to quantify the operative risk associated with small bowel and colorectal surgery in TX recipients, providing improved information on the actual surgical risk and identifying relevant risk factors in this specific population.

## 2 Methods

We retrospectively extracted data from our hospital's information system for all TX patients who underwent intestinal surgery at the University Hospital of Essen, Germany, from January 2008 to October 2024. This study was approved by the local ethics committee (24‐12246‐BO) and followed the Declaration of Helsinki. We included all consecutive patients with complete data to minimize selection and information bias.

Patient characteristics including sex, age, reason and date of TX, immunosuppression and comorbidities were recorded. Charlson Comorbidity Index (CCI) was assessed. Surgical procedures were categorized as small bowel and colorectal. Outcome was assessed by ICU‐stay, in‐hospital‐mortality, length of hospital stay (LOS) and morbidity (Dindo‐Clavien ≥ 3).

### 2.1 Statistics

Data were analyzed using SPSS 29.0 software (IBM Inc., Armonk NY, USA). A two‐sided T‐test was performed to compare mean values, and the 95%‐confidence interval (CI) was calculated. Continuous variables were analyzed on their original scale, data are presented as mean values with standard deviation or median and range as appropriate. Binary and linear logistic regression analysis were used to determine risk factors and other dependencies. Significant factors identified in the univariate analysis were included in the multivariate regression model. A *p*‐value of < 0.05 was considered statistically significant. Patients with missing data were excluded in the respective analysis.

## 3 Results

During the stated period, 237 transplant recipients underwent surgery at our center. Of these, 49.8% were male, and the mean age was 56.7 ± 19.4 years. Regarding comorbidities, the mean Charlson Comorbidity Index (CCI) was 5.46 ± 2.64. Additionally, the prevalence of diabetes and coronary heart disease was 26.6% and 17.3%, respectively. A total of 13.1% of patients had a diagnosis of carcinoma. The most frequent organ transplants were kidney (45.6%), liver (28.3%), and hematopoietic stem cell transplantation (HSCT) (13.1%). Mean time from transplantation to surgery was 8.14 ± 8.87 years. The majority of patients received prednisolone (70.5%), tacrolimus (65%), and/or mycophenolic acid (37.6%) as immunosuppressive agents. More detailed patients' characteristics are presented in Table 1.

**TABLE 1:** Patients' characteristics.

	*n* = 237
Sex (male/female)	119 (49.8%)/117 (50.2%)
Age (mean ± SD)	56.70 ± 19.4
CCI (mean ± SD)	5.46 ± 2.64
Coronary heart disease	41 (17.3%)
Diabetes	63 (26.6%)
Carcinoma	31 (13.1%)
Time from TX to surgery	8.14 ± 8.871 years
Kidney TX	108 (45.6%)
Heart TX	11 (4.6%)
Liver TX	67 (28.3%)
Lung TX	22 (9.3%)
Pancreas TX	5 (2.1%)
HSCT	31 (13.1%)
Multi TX	8 (3.4%)
Tacrolimus	154 (65.0%)
Prednisolone	167 (70.5%)
Mycophenolic acid	89 (37.6%)
Ciclosporin	34 (14.3%)
Everolimus	13 (5.5%)
Elective	65 (27.4%)
Emergency	172 (72.6%)
Small bowel	109 (46.0%)
Elective/emergency	17 (9.2%)/92 (90.8%)
Colorectal surgery	128 (54.0%)
Elective/emergency	48 (37.5%)/80 (62.5%)

Surgery was performed as an emergency in 72.6% of cases. 54% had colorectal surgery, 37.5% of these were elective. Primary anastomosis was performed in 84% of elective colorectal cases and 36% in emergency colorectal surgery. Indications for surgery are displayed in Table 2.

**TABLE 2:** Indications for surgery.

Indication		Small bowel	Colorectal
Obstruction	59 (24.7%)	51 (55%)	8 (6.3%)
Tumor	45 (18.8%)	11 (10.1%)	34 (26.6%)
Perforation	42 (17.6%)	11 (10.1%)	31 (24.2%)
Inflammatory	36 (15.1%)	12 (11.%)	24 (18.8%)
Ischemia	12 (5%)	5 (4.6%)	7 (5.5%)
Bleeding	9 (3.8%)	3 (2.8%)	6 (4.7%)
Hernia	8 (3.3%)	8 (7.3%)	—
Inflammtory bowel disease	2 (0.8%)	—	2 (1.6%)
Other	24 (19.6%)	8 (7.3%)	16 (11.7%)

The overall mortality rate was 20.3%, and the major morbidity rate was 47.7%. The mean length of stay (LOS) was 22 ± 28.5 days, and the mean intensive care unit (ICU) stay was 8.5 ± 18.5 days. Emergency surgery was associated with poorer outcomes across all assessed endpoints: mortality (25.6% vs. 6.2%, *p* = 0.001), morbidity (52.3% vs. 35.4%, *p* = 0.001), LOS (24.9 vs. 15.7 days, *p* = 0.005), and ICU stay (10.7 vs. 2.9 days, *p* = 0.001).

Colorectal surgery had an overall mortality of 25% and a morbidity of 46.9%, LOS 18.7 ± 17.8 days and ICU stays 8.0 ± 16.9 days. In elective cases mortality and morbidity were 8.3% and 35.4%, LOS 15.6 ± 12.7 and ICU stay 2.9 ± 6.8 days. Emergency colorectal surgery showed the poorest outcome in our cohort with a mortality of 35%, morbidity of 53.8% and respective LOS and ICU stay of 21.3 ± 21.0/11.1 ± 20.1 days.

Mortality and morbidity in small bowel surgery were 14.7% and 48.6% with mean LOS of 25.4 ± 36.2 days and ICU stay of 9.2 ± 20.4 days. In the few elective cases no patient died, morbidity was 35.3%. LOS and ICU stay were 16.2 ± 18.0 days and 3.2 ± 10.4, respectively. In emergency settings mortality was 17.4%, morbidity 51.1%, LOS and ICU stay 27.4 ± 38.8 and 10.3 ± 21.6, respectively.

**FIGURE 1:**
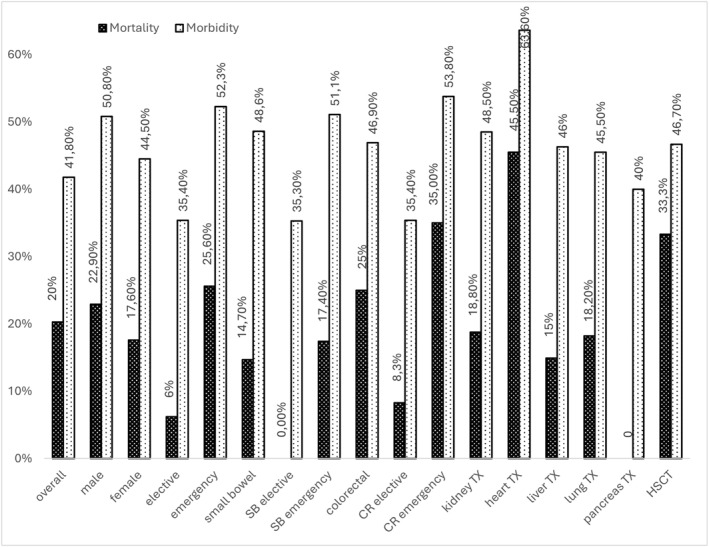
Outcomes in terms of mortality and morbidity sorted by sub groups. CR, colorectal; HSCT, hematopoetic stem cell transplant; SB, small bowel; TX, transplant.

Surgical procedure in terms of primary anastomosis had no influence on outcomes and surgical site infections. Patients with discontinuity resection had rather higher morbidity and mortality. For detailed analysis see Table 3 and Figure 1.

**TABLE 3:** Surgical procedures and outcome.

	*n*	Mortality	Morbidity	SSI
Discontinuity	57	23 (40.4%)	37 (64.9%)	11 (19.3%)
Anastomosis without diversion	107	14 (13.1%)	46 (43%)	16 (15%)
Anastomosis with diversion	9	1 (11%)	7 (77.8%)	2 (22%)
Small bowl				
Discontinuity	6	2 (33.3%)	4 (66.7%)	1 (16.7%)
Anastomosis without diversion	53	6 (11.3%)	27 (50.9%)	8 (15.1%)
Anastomosis with diversion	0			
Colorectal				
Discontinuity	51	21 (41.2%)	33 (64.7%)	10 (19.6%)
Anastomosis without diversion	54	8 (14.8%)	19 (35.2%)	8 (14.8%)
Anastomosis with diversion	9	1 (11.1%)	7 (77.8%)	2 (22.2%)
Colorectal elective				
Discontinuity	7	2 (28.6%)	4 (57.1%)	1 (14.3%)
Anastomosis without diversion	34	2 (5.9%)	11 (32.4%)	6 (17.6%)
Anastomosis with diversion	4	0	2 (50%)	1 (25%)
Colorectal emergency				
Discontinuity	44	19 (43.2%)	29 (65.9%)	9 (20.5%)
Anastomosis without diversion	20	6 (30%)	8 (40%)	2 (10%)
Anastomosis with diversion	5	1 (20%)	5 (100%)	1 (20%)

Heart transplant recipients experienced the worst outcomes (mortality 45.5%, morbidity 63.6%, LOS 25.8 days, ICU 22.1 days). An overview of patient outcomes is provided in Table 4. Further subgroups analysis for procedures and transplant types was performed, no significant results were reached under consideration of small sample size; trends were in line with the overall results for the larger combined groups.

**TABLE 4:** Patients' outcomes.

*N* = 237	Mortality	Morbidity	Surgical site infections	Cardio‐pulmonary complications	LOS	ICU stay
Overall	48 (20.3%)	130 (41.8%)	35 (14.8%)	23 (9.7%)	22.0 ± 28.5	8.5 ± 18.5
Male	27 (22.9%)	60 (50.8%)	24 (20.3%)	13 (11%)	21.3 ± 27.0	8.2 ± 16.5
Female	21 (17.6%)	53 (44.5%)	11 (9.2%)	10 (8.4%)	22.7 ± 30.0	8.7 ± 20.5
Elective	4 (6.2%)	23 (35.4%)	10 (15.4%)	4 (6.2%)	15.7 ± 14.1	2.9 ± 7.8
Emergency	44 (25.6%)	90 (52.3%)	25 (14.5%)	19 (11%)	24.9 ± 32.8	10.7 ± 20.9
Small bowel	16 (14.7%)	53 (48.6%)	15 (13.8%)	11 (10.1%)	25.4 ± 36.2	9.2 ± 20.4
Elective	0	6 (35.3%)	2 (11.8%)	2 (4.2%)	16.2 ± 18.0	3.2 ± 10.4
Emergency	16 (17.4%)	47 (51.1%)	12 (14.1%)	9 (9.8%)	27.4 ± 38.8	10.3 ± 21.6
Colorectal	32 (25.0%)	60 (46.9%)	20 (15.6%)	12 (9.4%)	18.7 ± 17.8	8.0 ± 16.9
Elective	4 (8.3%)	17 (35.4%)	8 (16.7%)	2 (4.2%)	15.6 ± 12.7	2.9 ± 6.8
Emergency	28 (35%)	43 (53.8%)	12 (15%)	10 (12.5%)	21.3 ± 21.0	11.1 ± 20.1
Kidney TX	19 (18.8%)	49 (48.5%)	19 (17.6%)	10 (9.3%)	21.4 ± 24.1	8 ± 17.6
Heart TX	5 (45.5%)	7 (63.6%)	1 (9.1%)	3 (27.3%)	25.8 ± 18.2	22.1 ± 40.3
Liver TX	10 (14.9%)	31 (46.3%)	9 (13.4%)	3 (4.5%)	17.2 ± 16.6	5.9 ± 10.1
Lung TX	4 (18.2%)	10 (45.5%)	2 (9.1%)	1 (4.5%)	15.2 ± 10.4	5.5 ± 8.8
Pancreas TX	0	2 (40%)	1 (20%)	1 (20%)	24.6 ± 29.0	10.4 ± 18.3
HSCT	10 (33.3%)	14 (46.7%)	4 (12.9%)	5 (16.1%)	40.8 ± 59.6	12.9 ± 25.5

To further evaluate morbidity, specific complications were assessed in detail. Surgical site infections (superficial, deep, and organ‐associated) occurred in 14.8% of all patients, cardiopulmonary complications (pulmonary embolism, pneumonia, etc.) in 9.7%, and major bleeding in 5.5%. No statistical accumulation of these specific complications was observed for the subgroups in our cohort.

Multivariate analysis for mortality revealed emergency surgery (OR 8.48 (2.68–26.8) *p* = 0.001), colorectal surgery (OR 3.26 (1.54–6.90) *p* = 0.002) and heart TX (OR 4.35 (1.10–17.23) *p* = 0.036) as independent risk factors, patients receiving prednisolone had reduced risk (OR 0.28 (0.13–0.60) *p* = 0.001). Regarding morbidity, emergency surgery (OR 1.98 (1.07–3.67) 0.03) was identified as risk factor. Just below the significance level diabetes could be noted as risk factor (OR 1.76 (0.95–3.25) *p* = 0.07) and prednisolone again with reduced risk (OR 0.57 (0.31–1.03) *p* = 0.06). Furthermore, multivariate linear regression analysis demonstrated that emergency surgery (coefficient 7.77 (2.55–12.84) *p* = 0.004) and heart transplantation (coefficient 11.39 (0.29–22.50) *p* = 0.04) were independent factors associated with longer ICU stay and HSCT with longer LOS (coefficient 17.57 (3.17–31.98) *p* = 0.017). The regression analysis was also performed for small bowel and colorectal surgery alone. Emergency surgery as risk factor and prednisolone with reduced risk could be reproduced for colorectal but not small bowel surgery. In the latter HSCT and mycophenolic acid were independent risk factors for mortality. Supporting Information S1: Table 1 displays the results of multivariate regression for the subgroups. The different indications for surgery such as inflammatory genesis, tumor or non‐malignant obstruction, were analyzed individually concerning outcome with no significant findings.

We could not always confirm whether an immunosuppressant switch was made preoperatively, as this often occurred at an external clinic. Our data reflect the actual immunosuppression regimen at the time of surgery. For elective cases, 66.2% of patients were on prednisolone, 69.2% on tacrolimus and 26.2% on mycophenolic acid, compared to 72.1% of emergency cases with prednisolone, 63.4% with tacrolimus and 41.9% on mycophenolic acid. The mean dose was 9.1 mg/day for elective cases and 13.2 mg/day for emergency cases (see Supporting Information S1: Table 2 for immunosuppressant overview). The reduced risk for prednisolone could be observed in both elective (OR 0.15 (0.015–1.5) *p* = 0.11, ns) and emergency setting (OR 0.38 (0.18–0.78) *p* = 0.01). Time from transplant to surgery was not a significant confounder (mortality OR: 1.005, CI: 0.97–1.04, *p* = 0.78; morbidity OR: 0.99, CI: 0.97–1.03, *p* = 0.96). In detailed perspective patients with a prednisolone dose more than 20 mg showed a trend toward worse outcome without reaching significance (mortality 24.5% vs. 12%, *p* = 0.07; Supporting Information S1: Table 3).

## 4 Discussion

The overall risk associated with surgery in transplant recipients is substantial. Even elective surgery carries a high risk of mortality (8.3%) and morbidity (35.4%), significantly elevated compared to the general population [21]. Similarly, the length of hospital stay (15.6 days) and ICU stay (2.9 days) are considerably longer than standard durations for colorectal surgery in non‐transplant patients [22]. These risks are markedly increased in emergency surgery within this population, which is also the primary risk factor in our cohort. Therefore, while acknowledging the heightened operative risk, surgery should not be avoided, as preventing the development of emergency situations is crucial. Notably, patients in this cohort presented with significant morbidity, indicated by a mean CCI of 5.46. Zhang and colleagues reported that a CCI ≥ 3 was already associated with a substantially increased risk of in‐hospital mortality following colorectal cancer surgery (OR 16.83, 95% CI [2.23–126.88], *p* = 0.0062) [23]. A case‐matched study on colorectal cancer in transplant patients demonstrated an increased risk associated with colorectal cancer surgery, even after adjusting for the CCI score [18].

Regression analysis allowed for the identification of specific risk factors beyond the general risks associated with immunosuppression. While the performance of surgery in emergency settings was an expected risk factor, the other findings warrant further investigation.

Considering comorbidities, the CCI did not show a further correlation with operative risk in our analysis. Despite our cohort's mean CCI of 5.26 and the reported increase in risk with a CCI greater than 3, we could not confirm a further increase in risk for higher CCI scores. Interestingly, transplant recipients not only have a significant risk of surgical site infections, but also a particular susceptibility to cardiopulmonary complications. While the risk of surgical site infections in transplant patients is well‐recognized in clinical practice, this elevated risk of cardiopulmonary complications has not yet received sufficient attention. An increase in cardiovascular events following transplantation has been described [24], but the underlying pathophysiology remains unclear and requires further investigation.

The less favorable outcomes observed with discontinuity resection may be attributed to its application in patients with more severe clinical presentations and therefore retrospective bias. Nevertheless, primary anastomosis in carefully selected TX patients does not elevate perioperative risk. This highlights the intricate nature of TX patients, where anastomotic integrity and potential surgical site infections are not the primary determinants of prognosis; rather, susceptibility to general and cardiopulmonary complications appears more critical. Consequently, we advocate for primary anastomosis in selected TX patients when technically feasible.

The poorer outcome in heart transplant recipients might be caused by the transplanted organ itself and its susceptibility to cardiopulmonary events. Transplant dysfunction in these patients often necessitates intensive care and monitoring, whereas dysfunctions of liver or kidney transplants can frequently be managed outside of an intensive care unit and are not necessarily life‐threatening. Heart TX patients are explicitly vulnerable in terms of perioperative risk. The longer mean hospital stay in HSCT patients may be caused by the considerably more complex transplantation, involving various confounding factors (e.g., malignancy, graft and donor origin, type of conditioning, or graft‐versus‐host disease) [6]. Interestingly, abdominal transplantations causing adhesions and altered anatomy might be more demanding for later surgery, but do not negatively influence outcome in comparison to thoracic transplantations or HCST in our cohort, underlining the perioperative risk in transplant patients is more of a systemic than a surgical origin.

Patients receiving prednisolone in our cohort exhibited a decreased risk of mortality and major morbidity. While acknowledging the inherent limitations of retrospective analyses and considering that the primary adverse effects of steroids are typically associated with long‐term use, perioperative steroid therapy may offer a protective effect. This could be attributed either to a direct benefit of steroids or to their potential role allowing the reduction of other, potentially more harmful immunosuppressants. Although prospective studies are warranted to confirm these findings, the clinical practice of reducing immunosuppressive agents, particularly mycophenolic acid, while increasing steroid dosage in elective surgery settings aligns with this observation. Still, prednisolone dosages above 20 mg appear to have a negative effect on surgical outcome, underlining the need of a critical approach.

The limitations of this study include the highly heterogeneous patient cohort, particularly regarding the surgical procedures performed. Furthermore, combining small bowel and colorectal surgery compounds this heterogeneity, which we addressed by structured subgroup stratification. Conversely, the strength of this study lies in its large cohort size, representing one of the largest single‐center cohorts of surgery in transplant recipients and, to our knowledge, the largest cohort specifically for small bowel and colorectal surgery in this population. Therefore, the statistical analysis, particularly the multivariate regression, yielded significant and robust results.

The data on the surgical risk of transplant recipients remains fragmented. While a generally increased risk is widely acknowledged, the actual morbidity and mortality rates are often unclear to treating physicians. This seemingly leads to apprehension when managing transplant patients, potentially resulting in suboptimal treatment decisions [20]. Further research on this topic and the involvement of transplant experts may improve clinical decision‐making.

## 5 Conclusions

Colorectal and small bowel surgery in transplant recipients is associated with considerable risks, which are markedly amplified in emergency settings. Notably, even elective colorectal surgery carries significant mortality (8.3%) and morbidity (35.4%), encompassing not only surgical site infections but also a substantial incidence (nearly 10%) of cardiopulmonary complications. Primary anastomosis can be considered in carefully selected patients. Furthermore, heart TX recipients face a demonstrably higher surgical risk compared to other organ transplant recipients. Optimal management necessitates the involvement of transplant experts to determine the indication and timing of surgery, as well as to consider a strategic perioperative shift toward steroid‐based immunosuppression, given their potentially favorable impact in this context.

## Author Contributions

**Phil Meister:** conceptualization, methodology, investigation, formal analysis, writing – original draft, writing – review and editing. **Samira Vestweber:** investigation, formal analysis. **Jan Neuhaus:** resources, data curation. **Marc A. Reschke:** conceptualization, methodology, validation, formal analysis. **Ulf Neumann:** supervision, resources, writing – review and editing. **Andreas D. Rink:** conceptualization, methodology, validation, supervision, resources, writing – review and editing.

## Funding

The authors have nothing to report.

## Ethics Statement

This study was approved by the local ethics committee (Medical Faculty, University of Duisburg‐Essen, 24‐12246‐BO) and followed the Declaration of Helsinki. No additional patient consent was required for retrospective analysis. The manuscript is in accordance with the STROBE statement. A preliminary version of this manuscript was presented as a poster at ESCP's 20th Scientific and Annual Conference, 10–12 September 2025, Paris, France.

## Conflicts of Interest

The authors declare no conflicts of interest.

## Supporting information


Supporting Information S1

## Data Availability

The datasets analyzed during the current study are not publicly available due to patient privacy limitations, but are available from the corresponding author on reasonable request.

## References

[1] A.‐K. Lederer , D. Haffa , V. Martini , et al., “Surgical Outcomes of Renal Transplant Recipients After Abdominal Surgery Not Connected With Transplantation. A Retrospective Case‐Control Study,” International Journal of Surgery 61 (2019): 53–59, 10.1016/j.ijsu.2018.12.002. 30540965

[2] D. Palamuthusingam , K. Kunarajah , E. M. Pascoe , D. W. Johnson , C. M. Hawley , and M. Fahim , “Postoperative Outcomes of Kidney Transplant Recipients Undergoing Non‐Transplant‐Related Elective Surgery: A Systematic Review and Meta‐Analysis,” BMC Nephrology 21, no. 1 (2020): 365, 10.1186/s12882-020-01978-4. 32843007 PMC7448361

[3] A. Savar , J. R. Hiatt , and R. W. Busuttil , “Acute Appendicitis After Solid Organ Transplantation,” Clinical Transplantation 20, no. 1 (2006): 78–80, 10.1111/j.1399-0012.2005.00444.x. 16556158

[4] A. Kilic , A. Sheer , A. S. Shah , S. D. Russell , C. G. Gourin , and A. O. Lidor , “Outcomes of Cholecystectomy in US Heart Transplant Recipients,” Annals of Surgery 258, no. 2 (2013): 312–317, 10.1097/sla.0b013e318287ab27. 23478523

[5] D. Gupta , G. H. Sakorafas , C. G. McGregor , W. S. Harmsen , and M. B. Farnell , “Management of Biliary Tract Disease in Heart and Lung Transplant Patients,” Surgery 128, no. 4 (2000): 641–649, 10.1067/msy.2000.108210. 11015098

[6] S. J. Bagley , A. R. Sehgal , S. Gill , et al., “Acute Cholecystitis Is a Common Complication After Allogeneic Stem Cell Transplantation and Is Associated With the Use of Total Parenteral Nutrition,” Biology of Blood and Marrow Transplantation 21, no. 4 (2015): 768–771, 10.1016/j.bbmt.2014.12.005. 25543093

[7] C. Blake , T. Almerey , D. Hyman , J. Nguyen , and J. A. Stauffer , “Pancreaticoduodenectomy After Liver Transplantation: A Single‐Center Experience,” World Journal of Surgery 47, no. 4 (2023): 1018–1022, 10.1007/s00268-022-06887-1. 36637476

[8] J. Mejia , I. Sucandy , J. Steel , et al., “Indications and Outcomes of Pancreatic Surgery After Liver Transplantation,” Clinical Transplantation 28, no. 3 (2014): 330–336, 10.1111/ctr.12317. 24757720

[9] A. Reshef , L. Stocchi , R. P. Kiran , et al., “Case‐Matched Comparison of Perioperative Outcomes After Surgical Treatment of Sigmoid Diverticulitis in Solid Organ Transplant Recipients Versus Immunocompetent Patients,” Colorectal Disease 14, no. 12 (2012): 1546–1552, 10.1111/j.1463-1318.2012.03077.x. 22564266

[10] S. Vaghiri , D. Prassas , W. T. Knoefel , and A. Krieg , “Surgical Management in Immunosuppressed Patients With Sigmoid Diverticulitis, Still a Challenge: A Single‐Center Observational Study,” International Journal of Colorectal Disease 37, no. 8 (2022): 1909–1917, 10.1007/s00384-022-04226-3. 35918442 PMC9388412

[11] J. T. Lee , S. Skube , G. B. Melton , et al., “Elective Colectomy for Diverticulitis in Transplant Patients: Is it Worth the Risk?,” Journal of Gastrointestinal Surgery: official journal of the Society for Surgery of the Alimentary Tract 21, no. 9 (2017): 1486–1490, https://pubmed.ncbi.nlm.nih.gov/28432506/. 28432506 10.1007/s11605-017-3432-z

[12] J. Sancho‐Muriel , H. Cholewa , M. Millán , et al., “Long‐Term Treatment Outcomes of Complicated Acute Diverticulitis in Immunocompromised Patients,” International Journal of Colorectal Disease 39, no. 1 (2024): 178, 10.1007/s00384-024-04753-1. 39496801 PMC11534823

[13] J. Ocaña , J. C. García‐Pérez , D. Fernández‐Martínez , et al., “Outcomes of Initially Nonoperative Management of Diverticulitis With Abscess Formation in Immunosuppressed Patients. DIPLICAB Study COLLABORATIVE Group,” Colorectal Disease 26, no. 1 (2024): 120–129, 10.1111/codi.16810. 38010046

[14] C. L. Klos , N. M. Bath , E. Carchman , et al., “Treating Acute Diverticulitis in Pre‐ and Post‐Solid‐Organ Transplant Patients: A Single‐Institution Cohort Study,” Colorectal Disease 25, no. 6 (2023): 1238–1247, https://pubmed.ncbi.nlm.nih.gov/36945080/. 36945080 10.1111/codi.16544

[15] F. Coccolini , M. Improta , M. Sartelli , et al., “Acute Abdomen in the Immunocompromised Patient: WSES, SIS‐E, WSIS, AAST, and GAIS Guidelines,” World Journal of Emergency Surgery 16, no. 1 (2021): 40, 10.1186/s13017-021-00380-1. 34372902 PMC8352154

[16] A. Merchea , F. Shahjehan , K. P. Croome , et al., “Colorectal Cancer Characteristics and Outcomes After Solid Organ Transplantation,” Journal of Oncology 2019 (2019): 5796108, 10.1155/2019/5796108. 30941176 PMC6421000

[17] M. E. D'Arcy , A. E. Coghill , C. F. Lynch , et al., “Survival After a Cancer Diagnosis Among Solid Organ Transplant Recipients in the United States,” Cancer 125, no. 6 (2019): 933–942, 10.1002/cncr.31782. 30624768 PMC6403005

[18] P. Meister , S. Vestweber , J. Neuhaus , M. A. Reschke , U. Neumann , and A. D. Rink , “Surgical Outcomes of Colorectal Cancer Surgery in Transplant Recipients: A Matched Case‐Control Study,” Colorectal Disease 27, no. 6 (2025): e70133, 10.1111/codi.70133. 40448302 PMC12125497

[19] E. Au , G. Wong , and J. R. Chapman , “Cancer in Kidney Transplant Recipients,” Nature Reviews Nephrology 14, no. 8 (2018): 508–520, 10.1038/s41581-018-0022-6. 29802400

[20] H. Benoni , C. Nordenvall , V. Hellström , et al., “Previous Solid Organ Transplantation Influences Both Cancer Treatment and Survival Among Colorectal Cancer Patients,” Transplant International: Official Journal of the European Society for Organ Transplantation 37 (2024): 13173, https://pubmed.ncbi.nlm.nih.gov/39371258/. 39371258 10.3389/ti.2024.13173 PMC11449720

[21] P. Baum , J. Diers , S. Lichthardt , et al., “Mortality and Complications Following Visceral Surgery: A Nationwide Analysis Based on the Diagnostic Categories Used in German Hospital Invoicing Data,” Deutsches Ärzteblatt International 116, no. 44 (2019): 739–746, https://pmc.ncbi.nlm.nih.gov/articles/PMC6912125/. 31774053 10.3238/arztebl.2019.0739 PMC6912125

[22] U. O. Gustafsson , M. J. Scott , M. Hubner , et al., “Guidelines for Perioperative Care in Elective Colorectal Surgery: Enhanced Recovery After Surgery (ERAS®) Society Recommendations: 2018,” World Journal of Surgery 43, no. 3 (2019): 659–695, 10.1007/s00268-018-4844-y. 30426190

[23] X. Zhang , X. Wang , M. Wang , et al., “Effect of Comorbidity Assessed by the Charlson Comorbidity Index on the Length of Stay, Costs, and Mortality Among Colorectal Cancer Patients Undergoing Colorectal Surgery,” Current Medical Research and Opinion 39, no. 2 (2023): 187–195, 10.1080/03007995.2022.2139053. 36269069

[24] M. H. Altieri , H. Liu , and S. S. Lee , “Cardiovascular Events After Liver Transplantation: MACE Hurts,” Reviews in Cardiovascular Medicine 23, no. 3 (2022): 91, 10.31083/j.rcm2303091. 35345258

