# Supplementary material for: Beyond Immunosuppression: Defining Perioperative Risk in Transplant Patients Undergoing Colorectal and Small Bowel Surgery
Source: World J Surg. 2026 Mar 4;50(4):1090–6. doi: 10.1002/wjs.70285 (PMC13070438; doi:10.1002/wjs.70285)
Supplement: Supplementary file 1 — Supporting Information S1 [file WJS-50-1090-s001.docx]

Supplementary Table 1: Overview of multivariate regression analysis for the overall cohort, small bowel and colorectal surgery. Only variables significant in multivariate regression for either cohort are displayed. Significant results are highlighted via bold font.

|  |  | |  | |  | |  | |  | |  |  |
| --- | --- | --- | --- | --- | --- | --- | --- | --- | --- | --- | --- | --- |
| Mortality | | Overall | |  | | Small Bowel | |  | | Colorectal | |  |
| Emergency | | **OR 8.48 (2.68-26.8)** | | **p=0.001** | | Not applicable | |  | | **OR 8.67 (2.28 – 33.0)** | | **p=0.002** |
| HTX | | **OR 4.35 (1.10-17.23)** | | **p=0.0036** | | OR 3.03 (0.26-35.57) | | p=0.38 | | OR 2.81 (0.88-14.4) | | p=0.19 |
| Prednisolon | | **OR 0.28 (0.13-0.60)** | | **p=.001** | | OR 0.55 (0.19-1.64) | | p=0.29 | | **OR 0.14 (0.04-0.43)** | | **p=<0.001** |
| HSCT | | OR 2.1 (0.9-4.83) | | p.08 | | **OR 4.99 (1.28-19.4)** | | **p=0.02** | | OR 1.36 (0.39-4.87) | | p=0.61 |
| MMF | | OR 1.38 (0.73-2.63) | | 0.32 | | **OR 4.63 (1.4-15.2)** | | **p=0.01** | | OR 0.68 (0.30-1.62) | | p=0.39 |

| Morbidity | Overall |  | Small Bowel |  | Colorectal |  |
| --- | --- | --- | --- | --- | --- | --- |
| Emergency | **OR 1.98 (1.07-3.67** | **p=0.003** | OR 1.92 (0.65-5.61) | p=0.24 | **OR 2.77 (1.13-6.80)** | **p=0.027** |
| Prednisolon | **OR 0.57 (0.31-1.03)** | **p=.06** | OR 1.67 (0.74-3.79) | p=0.22 | **OR 0.16 (0.06-0.49)** | **p=<0.001** |

Supplementary Table 2: Overview of immunosuppressant given for each transplant type.

|  | Tacrolimus | Prednisolon | Pred: mean dose | MMF | Everolimus | Ciclosporin |
| --- | --- | --- | --- | --- | --- | --- |
| Kidney TX | 67.6% | 87% | 10mg/d | 37% | 6.5% | 11.1% |
| Heart TX | 54.5% | 72.7% | 18mg/d | 54.5% | 0% | 36.4% |
| Liver TX | 77.6% | 41.8% | 12mg/d | 30% | 6% | 11.9% |
| Lung TX | 86.4% | 81.8% | 16mg/d | 77.3% | 4.5% | 5.8% |
| Pancreas TX | 100% | 100% | 7mg/d | 40% | 0% | 0% |
| HSCT | 19.4% | 67.7% | 15mg/d | 25.8% | 7.7% | 25.8% |

Supplementary Table 3: Outcome sorted by prednisolone dosage:

|  | Morbidity | Mortality |
| --- | --- | --- |
| >20mg, Overall | 51% | 24.5% |
| <20mg, Overall | 41% | 12% |
|  | p=0.23 | p=0.07 |
| >20mg, Colorectal | 48% | 27.6% |
| <20mg, Colorectal | 31% | 14.3% |
|  | p=0.17 | p=0.14 |
| >20mg, Smallbowel | 55% | 20% |
| <20mg, Smallbowel | 52% | 9% |
|  | p=0.81 | p=0.29 |
